# Improved Free Fatty Acid Production in Cyanobacteria with *Synechococcus* sp. PCC 7002 as Host

**DOI:** 10.3389/fbioe.2014.00017

**Published:** 2014-05-26

**Authors:** Anne M. Ruffing

**Affiliations:** ^1^Department of Bioenergy and Defense Technologies, Sandia National Laboratories, Albuquerque, NM, USA

**Keywords:** cyanobacteria, cyanobacterial biofuels, algal biofuels, free fatty acid, free fatty acid tolerance, *Synechococcus* sp. PCC 7002

## Abstract

Microbial free fatty acids (FFAs) have been proposed as a potential feedstock for renewable energy. The ability to directly convert carbon dioxide into FFAs makes cyanobacteria ideal hosts for renewable FFA production. Previous metabolic engineering efforts using the cyanobacterial hosts *Synechocystis* sp. PCC 6803 and *Synechococcus elongatus* PCC 7942 have demonstrated this direct conversion of carbon dioxide into FFAs; however, FFA yields in these hosts are limited by the negative impact of FFA production on the host cell physiology. This work investigates the use of *Synechococcus* sp. PCC 7002 as a cyanobacterial host for FFA production. In comparison to *S. elongatus* PCC 7942, *Synechococcus* sp. PCC 7002 strains produced and excreted FFAs at similar concentrations but without the detrimental effects on host physiology. The enhanced tolerance to FFA production with *Synechococcus* sp. PCC 7002 was found to be temperature-dependent, with physiological effects such as reduced photosynthetic yield and decreased photosynthetic pigments observed at higher temperatures. Additional genetic manipulations were targeted for increased FFA production, including thioesterases and ribulose-1,5-bisphosphate carboxylase/oxygenase (RuBisCO). Overexpression of non-native RuBisCO subunits (*rbcLS*) from a *psbAI* promoter resulted in more than a threefold increase in FFA production, with excreted FFA concentrations reaching >130 mg/L. This work illustrates the importance of host strain selection for cyanobacterial biofuel production and demonstrates that the FFA tolerance of *Synechococcus* sp. PCC 7002 can allow for high yields of excreted FFA.

## Introduction

Microbial production of free fatty acids (FFAs) has recently garnered much attention as a potential feedstock for renewable energy production (Handke et al., 2011; Lennen and Pfleger, 2012). Engineering efforts for FFA production in *Escherichia coli*, which began nearly four decades ago (Cronan et al., 1975), have increased substantially, leading to FFA yields as high as 5.1 g/L (Liu et al., 2012). However, FFA production from *E. coli* requires a fixed carbon source, such as glucose, for both microbial growth and FFA production. At present, these fixed carbon sources are prohibitively expensive to be used as feedstock for a low-value commodity such as fuel. While technology is currently under development to convert lignocellulosic biomass into a cost-effective carbon feedstock for biofuel production (Himmel et al., 2007), photosynthetic microorganisms offer an alternative means for FFA production, with the ability to directly convert carbon dioxide (CO_2_) into FFAs. Cyanobacteria are photosynthetic prokaryotes, which are particularly amenable to genetic manipulation, and as such, several cyanobacterial strains have been engineered for FFA production. *Synechocystis* sp. PCC 6803 and *Synechococcus elongatus* PCC 7942, two model freshwater strains of cyanobacteria, were engineered for FFA production by gene knockout of the FFA-recycling acyl-acyl carrier protein (ACP) synthetase/long-chain-fatty-acid CoA ligase and expression of a thioesterase for fatty acid (FA) cleavage from the ACP, resulting in extracellular FFA concentrations of 83.6 and 32 mg/L, respectively (Liu et al., 2011; Ruffing and Jones, 2012). In *Synechocystis* sp. PCC 6803, additional metabolic engineering strategies, such as deletion of polyhydroxybutyrate biosynthesis, overexpression of acetyl-CoA carboxylase, weakening of the peptidoglycan layer, and expression of thioesterases from different sources, yielded a maximum of 197 mg/L of excreted FFAs (Liu et al., 2011). Similarly, *S. elongatus* PCC 7942 was further modified by expression of an algal thioesterase, overexpression of ribulose-1,5-bisphosphate carboxylase/oxygenase (RuBisCO), expression of a chloroplastic acetyl-CoA carboxylase, and improved gene expression using strong native promoters, yet these modifications did not improve the concentration of excreted FFAs in engineered *S. elongatus* PCC 7942 (Ruffing, 2013a). These initial studies of cyanobacterial FFA production illustrate the feasibility of FFA biosynthesis directly from CO_2_ and also the difficulties associated with achieving high FFA yields using these cyanobacterial hosts.

In both *E. coli* and cyanobacteria, FFA production had detrimental effects on the cellular physiology of the host strain. These effects include reduced cell viability, compromised membrane integrity, and changes in membrane composition (Lennen et al., 2011), and the cyanobacterial hosts also had increased reactive oxygen species (ROS), reduced photosynthetic yields, and changes in photosynthetic pigments (Liu et al., 2011; Ruffing and Jones, 2012; Ruffing, 2013b). Interestingly, the change in membrane composition with FFA production varied depending on the host strain; *E. coli* had increased unsaturated membrane fatty acids while *S. elongatus* PCC 7942 showed higher levels of saturated membrane fatty acids during FFA production (Lennen et al., 2011; Ruffing and Jones, 2012). This discrepancy may be due to the differences in chain length of the synthesized FFAs for each host resulting from thioesterase selection, with *E. coli* producing C8-C14 FFAs and *S. elongatus* PCC 7942 producing mostly C16-C18 FFAs. Transcriptomics analyses of FFA production in both *E. coli* and *S. elongatus* PCC 7942 hosts revealed that significant changes in gene expression accompany FFA production. Both hosts had increased expression of genes involved in stress responses and energy production; however, specific gene responses were not conserved among these two hosts. The *E. coli* strains showed significant increases in phage shock response genes and genes involved in aerobic respiration (Lennen et al., 2011), while the *S. elongatus* PCC 7942 strains up-regulated genes involved in heat shock, ROS degradation, photosynthesis, electron transport, and nitrogen metabolism (Ruffing, 2013b). The different responses of *E. coli* and *S. elongatus* PCC 7942 are likely due to the fundamental differences in their carbon and energy metabolisms, i.e., glycolysis and aerobic respiration in *E. coli* vs. the Calvin–Benson–Bassham cycle and photosynthesis in *S. elongatus* PCC 7942. Regardless of the host-specific response, the physiological effects of FFA production are undesirable and likely limit the attainable FFA productivities of each host.

Due to the broad range of effects resulting from FFA biosynthesis and the variability in host cell response, alternative cyanobacterial hosts should be considered for FFA production. In this study, we investigate the use of *Synechococcus* sp. PCC 7002 for FFA production and excretion. Unlike the previous freshwater cyanobacterial strains used for FFA production, *Synechococcus* sp. PCC 7002 is a marine microorganism, capable of surviving salt concentrations as high as 1.7 M (Batterton and Baalen, 1971). As freshwater is an important resource, particularly for the non-arable lands proposed for biofuel production, marine hosts are necessary for economical large-scale biofuel production. Proposed microalgal biofuel production systems will also be exposed to outdoor environmental conditions, including high light intensities during peak sunlight as well as daily and seasonal temperature fluctuations. *Synechococcus* sp. PCC 7002 has desirable traits to address these concerns: *Synechococcus* sp. PCC 7002 displays extreme high light tolerance, surviving under light intensities as high as two times peak sunlight (4.5 mmol photons m^−2^ s^−1^) (Nomura et al., 2006), and it can survive under a wide range of temperatures, with high growth at 38°C (Ludwig and Bryant, 2012). This thermal tolerance is particularly important for growth in closed photobioreactors (PBRs), where temperatures will reach up to 40°C (Ong et al., 2010). The salt, light, and thermal tolerances of *Synechococcus* sp. PCC 7002 make it an ideal candidate for biofuel production, yet its tolerance for FFA production remains to be tested. This work investigated the FFA production capabilities and tolerances of *Synechococcus* sp. PCC 7002 in comparison to previously engineered strains of *S. elongatus* PCC 7942. FFA production was found to be less toxic in *Synechococcus* sp. PCC 7002 as compared to *S. elongatus* PCC 7942, yet this tolerance was temperature-dependent. Improved carbon fixation from RuBisCO overexpression in *Synechococcus* sp. PCC 7002 led to a threefold increase in FFA production, demonstrating a higher FFA yield than that achieved with comparably engineered strains of *S. elongatus* PCC 7942.

## Materials and Methods

### Materials

Chemicals used in this study were purchased from Acros Organics (Na_2_MoO_4_⋅2H_2_O), BD (agar), MP Biomedicals (CuSO_4_⋅5H_2_O), Fisher Scientific (NaCl, MgSO_4_⋅7H_2_O, KCl, NaNO_3_, tris base, H_3_BO_3_, and kanamycin monosulfate), and Sigma-Aldrich (Na_2_EDTA, CaCl_2_⋅2H_2_O, KH_2_PO_4_, vitamin B12, FeCl_3_⋅6H_2_O, MnCl_2_⋅4H_2_O, ZnCl_2_, CoCl_2_⋅6H_2_O, and spectinomycin dihydrochloride pentahydrate). Primers were synthesized by Integrated DNA Technologies (IDT). DNA purifications were performed using the Plasmid Miniprep, DNA Clean and Concentrator, and Gel DNA Recovery kits from Zymo Research. Restriction enzymes, DNA polymerases, and ligases were purchased from New England Biolabs. Suppliers of all other materials used in this study are described below.

### Strain construction

Strains used and constructed in this study are listed in Table 1. The first step in engineering *Synechococcus* sp. PCC 7002 for FFA production was gene knockout of the long-chain-fatty-acid CoA ligase (*fadD*, SYNPCC7002_A0675), responsible for FFA recycling (Kaczmarzyk and Fulda, 2010). Homologous regions (~500 bp) upstream and downstream of *fadD* were cloned using the primers in Table S2 in Supplementary Material and integrated into pSE15 (Ruffing and Jones, 2012) at the *Afl*II/*Spe*I (5′ fragment) and *Bgl*II/*Xho*I (3′ fragment) restriction enzyme sites, generating the *fadD* knockout plasmid, pS12. Transformation of pS12 into *Synechococcus* sp. PCC 7002, followed by spectinomycin selection and PCR screening of *fadD*, resulted in the construction of strain S01. Transformation of *Synechococcus* sp. PCC 7002 was performed using a modified protocol based on that proposed by Stevens and Porter (1980). *Synechococcus* sp. PCC 7002 was grown in medium A^+^ until OD_730_ = 1.0 was reached. Approximately, 0.1 μg of linearized plasmid DNA was added to 1 mL of culture (OD_730_ = 1.0) in a 16 mm glass test tube with ventilated cap. The transformation culture was incubated at 30°C with 150 rpm and 60 μmol photons m^−2^ s^−1^. After 24 h of incubation, the transformation culture was concentrated to 100 μL using centrifugation (5000 × *g*, 5 min) and spread on medium A^+^/agar plates with 1 mM of sodium thiosulfate and 40 μg/mL of spectinomycin dihydrochloride pentahydrate and 50 μg/mL of kanamycin monosulfate, as required. Colonies were re-streaked a minimum of three times to obtain complete segregants. After antibiotic selection and PCR screening, two positive transformants were tested for FFA production in 400 mL cultures (see Culture Conditions).

**Table 1:** **Strains used and constructed in this study**.

Strain	Description	Reference
*Escherichia coli* DH5α	*E. coli* strain used for cloning and plasmid construction	New England Biolabs
*Synechococcus elongatus* PCC 7942	Model freshwater cyanobacterium (ATCC 33912); previously used as host for FFA biosynthesis (Ruffing and Jones, 2012; Ruffing, 2013a,b)	American type culture collection
*Synechococcus* sp. PCC 7002	Model marine cyanobacterium (ATCC 27264); host for FFA biosynthesis	American type culture collection
SE01	*S. elongatus* PCC 7942 with gene knockout of the acyl-ACP synthetase/long-chain-fatty-acid CoA ligase (*aas*)	Ruffing and Jones (2012)
SE02	*S. elongatus* PCC 7942 with gene knockout of the acyl-ACP synthetase/long-chain-fatty-acid CoA ligase (*aas*) and expression of truncated *E. coli* thioesterase (*‘tesA*)	Ruffing and Jones (2012)
SE03	*S. elongatus* PCC 7942 with gene knockout of the acyl-ACP synthetase/long-chain-fatty-acid CoA ligase (*aas*) and expression of *C. reinhardtii* acyl-ACP thioesterase (*fat1*)	Ruffing (2013a)
SE04	*S. elongatus* PCC 7942 with gene knockout of the acyl-ACP synthetase/long-chain-fatty-acid CoA ligase (*aas*), expression of *C. reinhardtii* acyl-ACP thioesterase (*fat1*), and expression of *rbcLS* (P_trc_-*fat1-rbcLS*)	Ruffing (2013a)
SE06	*S. elongatus* PCC 7942 with gene knockout of the acyl-ACP synthetase/long-chain-fatty-acid CoA ligase (*aas*), expression of *C. reinhardtii* acyl-ACP thioesterase (*fat1*), and expression of RuBisCO (*rbcLS*) from the *psbAI* promoter of *S. elongatus* PCC 7942 (P_trc_-*fat1*-P*_psbAI_*-*rbcLS*)	Ruffing (2013a)
S01	*Synechococcus* sp. PCC 7002 with gene knockout of the acyl-ACP synthetase/long-chain-fatty-acid CoA ligase (*fadD*)	This study
S02	*Synechococcus* sp. PCC 7002 with gene knockout of the acyl-ACP synthetase/long-chain-fatty-acid CoA ligase (*fadD*) and expression of truncated *E. coli* thioesterase (*‘tesA*)	This study
S02Δ*desB*	*Synechococcus* sp. PCC 7002 with gene knockout of the acyl-ACP synthetase/long-chain-fatty-acid CoA ligase (*fadD*) and desaturase B (*desB*), along with expression of truncated *E. coli* thioesterase (*‘tesA*)	This study
S03	*Synechococcus* sp. PCC 7002 with gene knockout of the acyl-ACP synthetase/long-chain-fatty-acid CoA ligase (*fadD*) and expression of *C. reinhardtii* acyl-ACP thioesterase (*fat1*)	This study
S05	*Synechococcus* sp. PCC 7002 with gene knockout of the acyl-ACP synthetase/long-chain-fatty-acid CoA ligase (*fadD*) and expression of a truncated *C. reinhardtii* acyl-ACP thioesterase (*tfat1*)	This study
S06	*Synechococcus* sp. PCC 7002 with gene knockout of the acyl-ACP synthetase/long-chain-fatty-acid CoA ligase (*fadD*), expression of truncated *E. coli* thioesterase (*‘tesA*), and expression of RuBisCO (*rbcLS*) (P_trc_-*‘tesA*-*rbcLS*)	This study
S07	*Synechococcus* sp. PCC 7002 with gene knockout of the acyl-ACP synthetase/long-chain-fatty-acid CoA ligase (*fadD*), expression of truncated *E. coli* thioesterase (*‘tesA*), and expression of RuBisCO (*rbcLS*) from the *psbAI* promoter of *S. elongatus* PCC 7942 (P_trc_-*‘tesA*-P*_psbAI_*-*rbcLS*)	This study

For S02 construction, the truncated *E. coli* thioesterase, *‘tesA*, from pSE16 (Ruffing and Jones, 2012) was cloned using primers in Table S2 in Supplementary Material and integrated into pS12 at the *Eco*RI and *Bam*HI sites following the inducible trc promoter to form pS13. Transformation of pS13 into *Synechococcus* sp. PCC 7002 resulted in gene knockout of *fadD* and integration of *‘tesA*. Gene knockout of the desaturase gene, *desB*, was achieved by transforming pSB into S02. pSB was constructed by replacing the NSII homologous regions of pSA (Ruffing, 2013a) with 951 and 936 bp fragments homologous to the sequences upstream and downstream of *desB*. The *fat1* thioesterase from *Chlamydomonas reinhardtii* was cloned from pSE20 and integrated at the *Eco*RI and *Bam*HI sites of pS12 to form pS14, using primers listed in Table S2 in Supplementary Material. Strain S03 was constructed by transformation and integration of pS14 in *Synechococcus* sp. PCC 7002. A truncated *fat1* thioesterase (*tfat1*) was constructed by redesigning the forward primer to eliminate the predicted chloroplast-targeting signal, determined using ChloroP 1.1 (Emanuelsson et al., 1999). Integration of *tfat1* into the *Eco*RI and *Bam*HI sites of pS12 yielded pS17, and subsequent transformation and integration of pS17 into *Synechococcus* sp. PCC 7002 produced strain S05.

To insert another restriction enzyme site for *rbcLS* integration, *‘tesA* was re-cloned using primer ‘tesAR2, yielding pS18. The small and large subunits of RuBisCO (*rbcLS*) from *S. elongatus* PCC 7942 were cloned from pSE18 (Ruffing, 2013a) (primers rbcLSF1/rbcLSR1 in Table S2 in Supplementary Material) and inserted downstream of *‘tesA* at the *Aat*II site in pS18 to produce pS19. Transformation of pS19 into *Synechococcus* sp. PCC 7002 yielded strain S06. To insert the *psbA1* promoter from *S. elongatus* PCC 7942 upstream of *rbcLS*, the *psbA1* promoter was cloned from pSE20 and inserted into pS18 to yield pS20. Subsequent cloning of *rbcLS* from SE20 using the second *rbcLS* primer set (rbcLSF2/rbcLSR2) and insertion into pS20 at the *Bsr*GI site produced pS21. Transformation of pS21 into *Synechococcus* sp. PCC 7002 yielded strain S07.

*Escherichia coli* DH5α was used for plasmid construction; competent cells were prepared as described in Sambrook and Russell (2001). All *E. coli* DH5α strains used for plasmid maintenance were grown in LB medium at 37°C and 350 rpm in a VWR incubating mini-shaker. After overnight growth in a test tube containing 4 mL of LB media, the plasmid-containing *E. coli* strains were harvested for plasmid isolation using the plasmid mini-prep kit from Zymo Research. Plasmids derived from pS12 were linearized using *Nde*I digestion, and plasmids derived from pSA were linearized using *Spe*I digestion.

### Culture conditions

Wild-type and engineered strains of *Synechococcus* sp. PCC 7002 were grown in medium A^+^ with antibiotic supplementation as required (40 μg/mL spectinomycin dihydrochloride pentahydrate for S01, S02, S02Δ*desB*, S03, S05, S06, and S07 and 50 μg/mL of kanamycin monosulfate for S02Δ*desB*). Agar plates of medium A^+^ contained 1 mM of sodium thiosulfate. Single transformed colonies were re-streaked at least three times or until full segregants were obtained, as confirmed by PCR. *Synechococcus* sp. PCC 7002 strains grown on solid media were inoculated into 4 mL of medium A^+^ with antibiotics as necessary and grown at 30 or 38°C and 150 rpm in a New Brunswick Innova 42R shaking incubator with photosynthetic light bank. Alternating cool white and plant fluorescent lights provided ~60 μmol photons m^−2^ s^−1^ of continuous illumination. After 5–7 days of growth, 1 mL of the test tube inoculum was transferred to a 500 mL baffled Erlenmeyer flask containing 100 mL of medium A^+^ with appropriate antibiotics. After 4 days of growth under the aforementioned conditions, the 100 mL culture was used to inoculate 400 mL of medium A^+^ in a 1 L glass media bottle with a three-port lid for sampling, bubbling of 1% CO_2_ in air, and ventilation through a 0.22 μm filter. The initial cell concentration of the 400 mL culture vessels was approximately an OD_730_ of 0.1. No antibiotics were added to the large culture vessels to eliminate any potential effects associated with antibiotic supplementation. The large cultures were sampled every 2 days for ~3 weeks. IPTG was added at 100 h to induce expression from the trc and LlacO-1 promoters.

### Physiological measurements

Cell growth was measured using optical density (OD) readings at 730 nm from a PerkinElmer Lambda Bio spectrophotometer. Photosynthetic yield measurements were obtained using a Waltz mini-PAM photosynthetic yield analyzer; for each sample, triplicate technical measurements were averaged due to variability associated with these measurements. Photosynthetic yield or efficiency measurements $\left(F_{\text{V}}^{\prime }∕F{\;}_{\text{m}}^{\prime }\right)$ are a fluorescence-based measurement of the photochemical efficiency of photosystem II (PSII) in higher plants. Variable fluorescence (*F*_v_) is the difference between the maximum fluorescence (*F*_m_) under saturated light and the initial fluorescence (*F*_0_) under actinic light; the apostrophe indicates that the samples were not dark adapted prior to measurement. Photosynthetic yield $\left(F_{\text{V}}^{\prime }∕F{\;}_{\text{m}}^{\prime }\right)$ measurements in cyanobacteria are complicated by fluorescence contributions from phycobiliproteins and the low fraction of total chlorophyll associated with PSII; however, $\left(F_{\text{V}}^{\prime }∕F{\;}_{\text{m}}^{\prime }\right)$ measurements in cyanobacteria have been shown to correlate well with changes in the rate of oxygen evolution from PSII (Campbell et al., 1998). In this study, photosynthetic yield measurements are used as a general indicator of photosynthetic efficiency and overall cell health. FFA measurements were performed as described previously (Ruffing and Jones, 2012) using the FFA Quantification Kit from Biovision. Cell growth, photosynthetic yield, and FFA measurements were taken every 2 days. At day 16, the absorbance spectrum of each culture was measured using a Beckman Coulter DU-800 UV-Vis Spectrophotometer to determine changes in photosynthetic pigment concentrations. Each data point is the average of three biological replicates with error bars indicating the standard deviation.

## Results

### Reduced FFA toxicity in *Synechococcus* sp. PCC 7002 compared to *S. elongatus* PCC 7942

**Figure 1:**
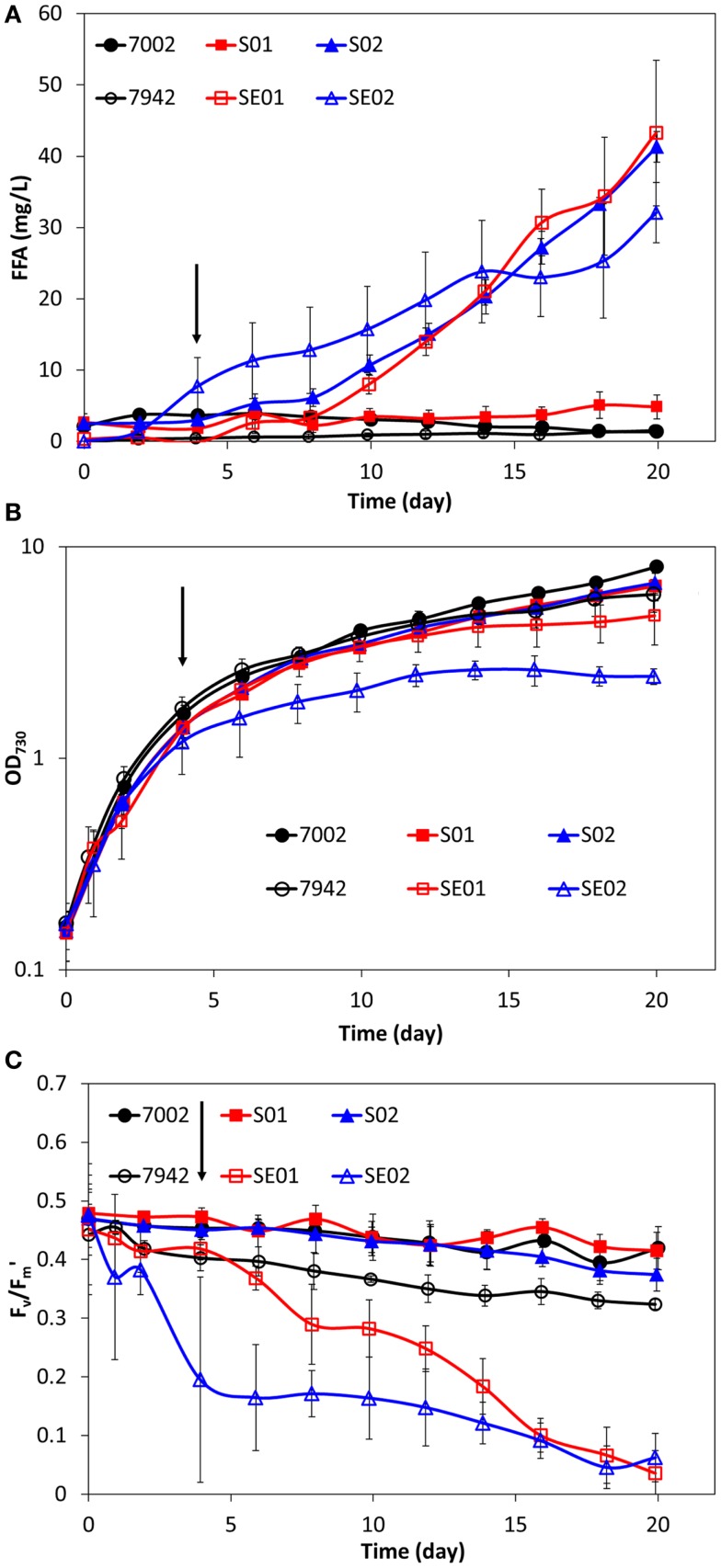
**Comparison of extracellular FFA concentration (A), cell concentration (B), and photosynthetic yields (C) during FFA production in two cyanobacterial hosts: *S. elongatus* PCC 7942 and *Synechococcus* sp. PCC 7002**. Wild-type strains are illustrated with black circles (7942 – open, 7002 – filled). Strains with gene knockout of the FFA-recycling acyl-ACP synthetase/long-chain-fatty-acid CoA ligase (*aas*/*fadD*) are illustrated with red squares (SE01 – open, S01 – filled). Strains with gene knockout of the FFA-recycling gene and *‘tesA* expression are illustrated with blue triangles (SE02 – open, S02 – filled). Addition of IPTG is indicated by the arrows (100 h). All data are averages of at least three biological replicates with error bars indicating the standard deviation.

*Synechococcus* sp. PCC 7002 was genetically engineered for FFA production and excretion by targeting the long-chain-fatty-acid CoA ligase (*fadD*, SYNPCC7002_A0675) for gene knockout and overexpression of a truncated thioesterase from *E. coli* (*‘tesA*). The resulting engineered strains, S01 (Δ*fadD*) and S02 (Δ*fadD, ‘tesA*^+^), are analogous to the previously constructed, FFA-producing strains of *S. elongatus* PCC 7942, SE01 (Δ*aas*) and SE02 (Δ*aas, ‘tesA*^+^). A schematic of the engineered pathway for FFA production can be found in the Supplemental Material (Figure S1). The FFA concentrations, cell growth profiles, and photosynthetic yields of *Synechococcus* sp. PCC 7002, S01, and S02 are shown in Figure 1, along with the previous results from *S. elongatus* PCC 7942, SE01, and SE02 (Ruffing and Jones, 2012). As expected, no FFAs are excreted by the wild-type (7002), yet both S01 and S02 synthesized and excreted FFAs. The FFA concentrations produced by *fadD* knockout in S01 (Figure 1A) are very low (<6 mg/L), particularly in comparison to the comparably engineered *S. elongatus* PCC 7942 strain, SE01, which produced up to 43 mg/L of excreted FFAs. Expression of the truncated *E. coli* thioesterase in S02, however, increased the level of excreted FFAs, producing concentrations similar to SE01 and higher levels than the analogous *S. elongatus* PCC 7942 strain, SE02 (Figure 1A). While the FFA-producing strains of *Synechococcus* sp. PCC 7002 (S01 and S02) did not show improved FFA production in comparison to the *S. elongatus* PCC 7942 strains (SE01 and SE02), there were notable differences in the physiological responses of the hosts. The late exponential growth rates of S01 and S02 showed a slight decrease in comparison to the wild-type 7002 after induction at 100 h (4.17 days) (Figure 1B); this small reduction is expected as FFA production reduces the available pool of acyl-ACP for cell membrane biosynthesis. On the other hand, SE01 and SE02 showed a severe reduction in final cell concentrations, 21 and 59% lower than wild-type (Figure 1B), much greater than expected due to FFA production. Additionally, the photosynthetic yields of S01 and S02 are very similar to the wild-type 7002, while the photosynthetic yields of SE01 and SE02 were significantly reduced compared to the wild-type 7942 strain (Figure 1C). These results suggest that FFA production in *Synechococcus* sp. PCC 7002 does not compromise the cellular physiology of the host strain, unlike the engineered strains of *S. elongatus* PCC 7942, which exhibited a stress response to FFA production and excretion.

### FFA tolerance in *Synechococcus* sp. PCC 7002 is temperature-dependent

**Figure 2:**
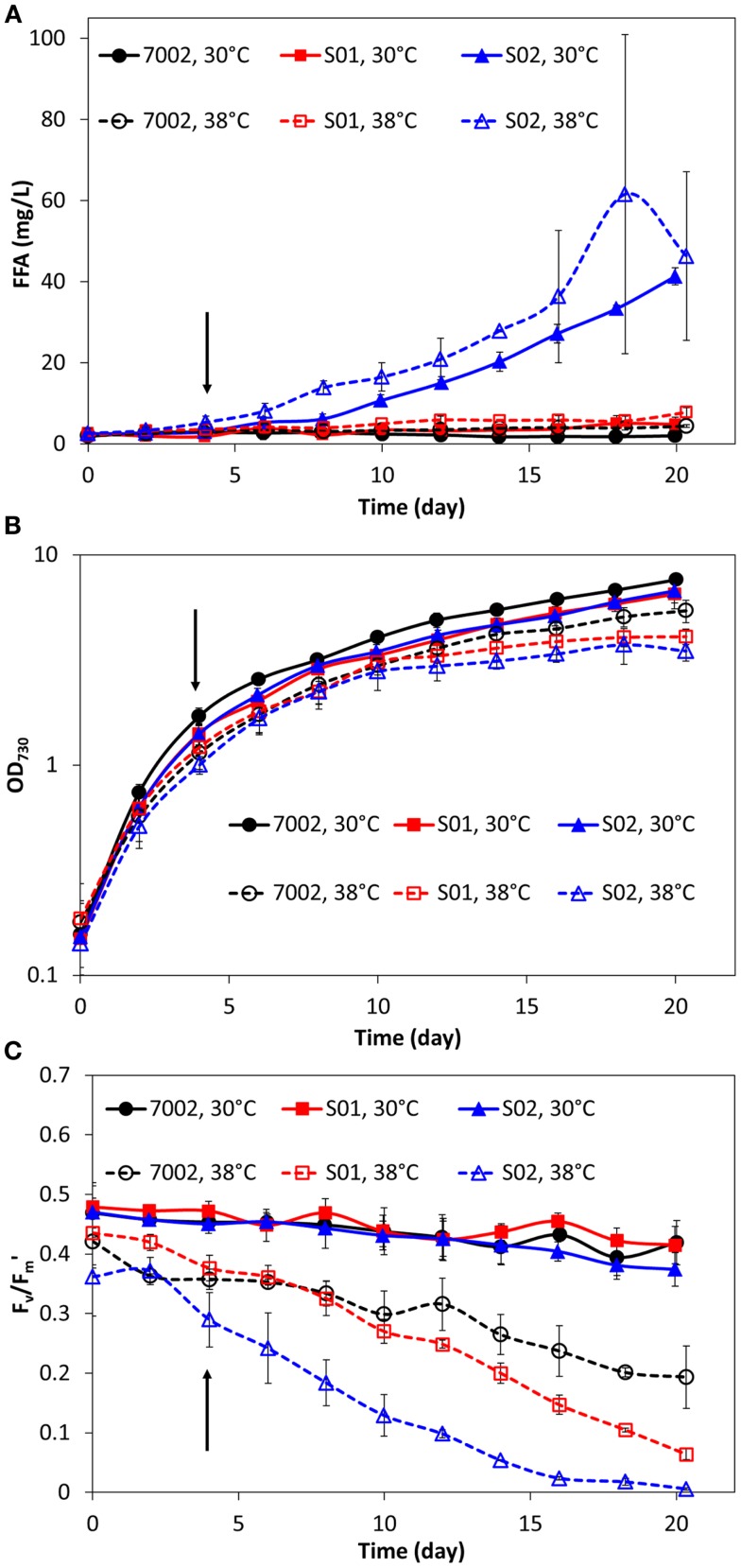
**Comparison of extracellular FFA concentration (A), cell concentration (B), and photosynthetic yields (C) during FFA production in *Synechococcus* sp. PCC 7002 strains at two temperatures: 30°C (solid lines, filled markers) and 38°C (dashed lines, open markers)**. Addition of IPTG is indicated by the arrows (100 h). All data are averages of at least three biological replicates with error bars indicating the standard deviation.

**Figure 3:**
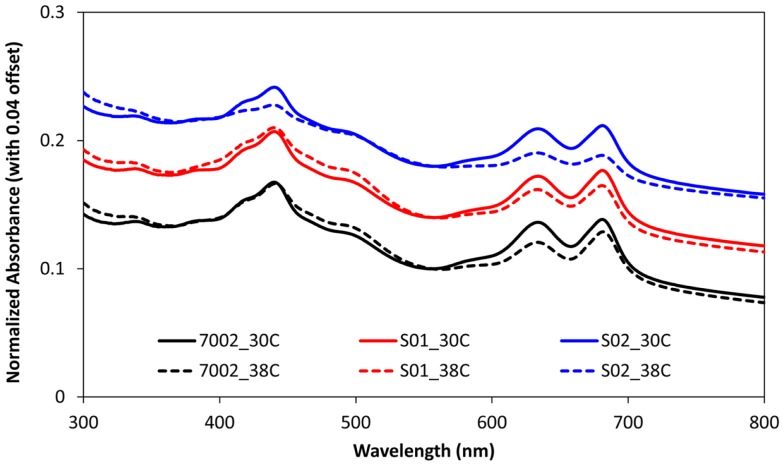
**Absorbance spectra of *Synechococcus* sp. PCC 7002 strains at day 16 for 30°C (solid lines) and 38°C (dashed lines) conditions**. OD readings were normalized with respect to OD_556_ to account for variations in cell density, and spectra for each strain were offset by 0.04 to aid in visualization. The phycobiliproteins, phycocyanin, and allophycocyanin, have a peak at 635 nm, and chlorophyll-*a* pigments have a peak at 680 nm with Soret bands at 440 nm.

The FFA production experiments described in the previous section and the results reported in Figure 1 were conducted at a temperature of 30°C for comparison with the engineered *S. elongatus* PCC 7942 strains, which have an optimal growth temperature of 30°C (Golden and Sherman, 1984). However, the reported optimal growth temperature for *Synechococcus* sp. PCC 7002 is 38°C (Sakamoto and Bryant, 1997). Therefore, FFA production experiments were also conducted at this higher temperature for 7002, S01, and S02 (Figure 2). As expected, the higher cultivation temperature yielded a slight improvement in FFA concentrations excreted by S01 and S02 (Figure 2A), likely due to the increased thermal kinetics associated with the higher temperature. Unexpectedly, cell growth was reduced at the higher temperature for all three strains, including the wild-type (Figure 2B). To confirm the culture temperature, the aqueous temperature of the culture vessels was measured using a standard glass thermometer. The aqueous temperature readings indicated that the culture temperatures were slightly higher than the setpoints, 32°C for the 30°C setting and 39°C for the 38°C setting. The lower range light intensities used in this experiment (60 μmol photons m^−2^ s^−1^) may also influence the optimal growth temperature, and a more rigorous, multi-factorial investigation of *Synechococcus* sp. PCC 7002 growth is required to evaluate the optimal growth temperature of this strain. In addition to this unexpected growth response, there were significant changes in photosynthetic yields at 38°C in comparison to 30°C. At the higher temperature, the wild-type (7002) had reduced photosynthetic yields with values up to 50% lower compared to the 30°C temperature condition (Figure 2C). The FFA-producing strains showed an additional decrease in photosynthetic yield, with S02, the highest FFA producer, having the most reduced photosynthetic yields. To investigate whether these reduced photosynthetic yields were accompanied by changes in photosynthetic pigments, the absorbance spectra of the wild-type (7002) and engineered strains (S01 and S02) were measured for cultures at 38 and 30°C after significantly reduced photosynthetic yields were detected (day 16). While the absorbance spectrum for the wild-type (7002) did not show significant differences at the two temperatures, the spectrum of the S02 cultures showed reduced peaks for both chlorophyll-*a* (680 nm) and the phycobiliproteins (635 nm) at 38°C (Figure 3). The reduced growth, photosynthetic yields, and photosynthetic pigments that accompany FFA production at 38°C suggest that the FFA tolerance of *Synechococcus* sp. PCC 7002 is temperature-dependent.

At high temperatures, bacteria have been shown to change the degree of saturation of their cell membrane lipids, with increasing saturation accompanying increasing temperatures (Koga, 2012). The increased saturation of membrane FAs in *Synechococcus* sp. PCC 7002 at 38°C may affect the ability of FFAs to cross the cell membrane. Alternatively, the removal of saturated FAs by the recombinant thioesterase may compromise the cell’s ability to grow at this higher temperature. To investigate this hypothesis, the S02 strain was modified via gene knockout of the *desB* gene, encoding a desaturase involved in cold temperature tolerance in *Synechococcus* sp. PCC 7002 (Sakamoto et al., 1997). At 30°C, the *desB* mutant did not show any significant changes in FFA production, growth, or photosynthetic yield in comparison to S02 (data not shown). This suggests that DesB is not involved in FFA tolerance at this temperature, but it does not rule out a role for membrane saturation in the mechanism of temperature-dependent FFA tolerance in *Synechococcus* sp. PCC 7002.

### Genetic targets for improved FFA production

**Figure 4:**
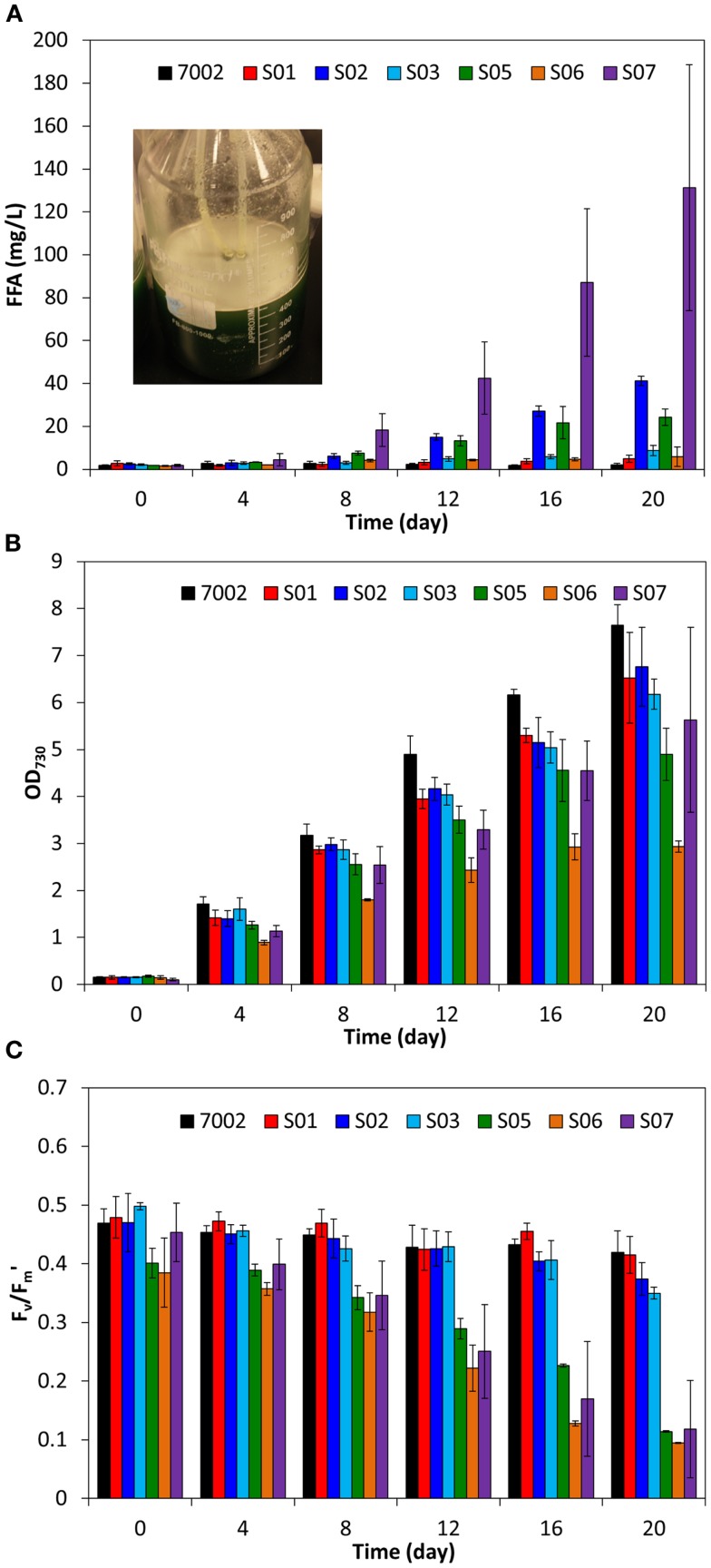
**Comparison of extracellular FFA concentration (A), cell concentration (B), and photosynthetic yields (C) during FFA production in engineered *Synechococcus* sp. PCC 7002 strains**. The inset image in plot A shows FFA precipitation and floatation (white precipitate) in S07 cultures after 20 days. All data are averages of at least three biological replicates with error bars indicating the standard deviation.

To determine if higher yields of FFA can be produced in *Synechococcus* sp. PCC 7002 compared to *S. elongatus* PCC 7942, additional strategies for improving FFA biosynthesis were investigated. The acyl-ACP thioesterase from *C. reinhardtii* (*fat1*) was previously shown to have activity similar to *‘tesA* in *S. elongatus* PCC 7942 (Ruffing, 2013a). Therefore, *fat1* was expressed in *Synechococcus* sp. PCC 7002, along with *fadD* knockout, to form strain S03. Unexpectedly, S03 showed only a slight increase in excreted FFA concentration compared to S01, which has only the *fadD* knockout (Figure 4A). The *fat1* gene was previously cloned from the associated *C. reinhardtii* mRNA transcript (Ruffing, 2013a), which likely includes a chloroplast-targeting signal for translocation of Fat1, a nuclear-encoded protein, into the chloroplast. Inclusion of the chloroplast-targeting signal may lead to either low enzyme activity or secretion of the enzyme, as the cyanobacterial membrane is evolutionarily similar to the chloroplast membrane (Giovannoni et al., 1988). To eliminate this potential source of low activity, a putative chloroplast-targeting signal was predicted using ChloroP 1.1 (Emanuelsson et al., 1999), and a new forward primer was designed to exclude this 5′ signal sequence, yielding a truncated *fat1* (*tfat1*). Expression of *tfat1* in S05 led to a nearly threefold increase FFA production and excretion compared to the *fat1*-expressing S03 strain (Figure 4A). However, the amount of FFA produced by S05 was still lower than that produced by the *‘tesA*-expressing S02 strain. Additionally, S05 had lower growth and photosynthetic yield compared to S02 (Figures 4B,C); therefore, further strain development utilized the *‘tesA* thioesterase rather than *fat1* or *tfat1* acyl-ACP thioesterases.

In addition to thioesterase expression, carbon fixation by RuBisCO is an important step for high carbon flux to support both growth and FFA biosynthesis. Similar to RuBisCO overexpression in *S. elongatus* PCC 7942, expression of the large and small subunits of RuBisCO from *S. elongatus* PCC 7942 (*rbcLS*) did not yield an improvement in FFA production in the *Synechococcus* sp. PCC 7002 strain S06 [(Ruffing, 2013a), Figure 4A]. Previous quantitative PCR studies in engineered *S. elongatus* PCC 7942 indicated that *rbcLS* expression is not significantly improved by integration of the P_trc_-*fat1-rbcLS* synthetic operon (Ruffing, 2013a). Therefore, an additional promoter was inserted into the synthetic operon to drive *rbcLS* expression. The *psbA1* promoter from *S. elongatus* PCC 7942 was cloned and inserted after *‘tesA* to form the synthetic operon: P_trc_-*‘tesA*-P*_psbA1_*-*rbcLS* in S07, an engineered strain of *Synechococcus* sp. PCC 7002. Contrary to the results obtained in the *S. elongatus* PCC 7492 host (Ruffing, 2013a), *rbcLS* overexpression in S07 resulted in more than a threefold increase in excreted FFA concentrations (Figure 4A), suggesting that carbon fixation is in fact a rate-limiting step in FFA biosynthesis for *Synechococcus* sp. PCC 7002. The extracellular FFA concentrations in the S07 cultures had high variability, as illustrated by the error bars in Figure 4A, which represent the standard deviation of the three biological replicates. This measurement variability was due to FFA precipitation, illustrated by the inset image in Figure 4A, which made homogenous sampling difficult. The higher FFA production in S07 also resulted in reduced cell growth and photosynthetic yields (Figures 4B,C), indicating that high levels of FFA production may affect cellular physiology in *Synechococcus* sp. PCC 7002.

## Discussion

The results presented in this work demonstrate that *Synechococcus* sp. PCC 7002 has a higher tolerance for FFA production compared to the freshwater cyanobacterium, *S. elongatus* PCC 7942. As such, *Synechococcus* sp. PCC 7002 is a beneficial host for photosynthetic FFA biosynthesis. The FFA tolerance of *Synechococcus* sp. PCC 7002 was shown to be dependent on both temperature and FFA concentration, as high temperature or high FFA production compromised the physiology of the host cell. Through gene knockout of the acyl-ACP synthetase/long-chain-fatty-acid CoA ligase (*fadD*), overexpression of the truncated *E. coli* thioesterase (*‘tesA*), and overexpression of a heterologous RuBisCO (*rbcLS*), an engineered strain of *Synechococcus* sp. PCC 7002 was constructed, which produced FFAs at concentrations as high as 131 mg/L. The high levels of FFA achieved in this study demonstrate not only a potential for large-scale production applications, but also a potential separation mechanism, as the FFAs precipitated from the culture medium and floated to the surface upon reaching saturation (Figure 4A, inset).

Surprisingly, *Synechococcus* sp. PCC 7002 showed higher FFA tolerance at a low growth temperature of 30°C rather than its reported optimum of 38°C. Temperature-induced changes in the chemical composition of the cell membrane may be responsible for the low FFA tolerance of *Synechococcus* sp. PCC 7002 at higher temperatures. The increase in saturated membrane FAs at higher temperatures may influence the ability of FFAs to pass through the cell membrane, ultimately leading to intercalation of the FFAs and membrane damage. Alternatively, expression of the recombinant thioesterase may lead to a reduced level of available saturated FAs for membrane biosynthesis, compromising the cell’s ability to grow at the elevated temperature. High temperatures will also influence the solubility of FFAs, with a positive correlation between FFA solubility and temperature (Oliveira et al., 2009). Thus, the enhanced FFA solubility at 38°C will increase the soluble FFA fraction, leading to higher effective FFA concentrations in the culture. In this study as well as previous work, we have shown that high concentrations of FFAs will lead to detrimental effects on cell growth and physiology. The FFAs may compromise cellular integrity via integration into the cellular membranes and disruption of the activity of membrane-associated enzymes, such as those involved in photosynthesis. While the mechanism of this temperature-dependent FFA tolerance remains to be elucidated, the physiological effects of FFA production can be alleviated in *Synechococcus* sp. PCC 7002 by using lower temperatures (near 30°C) which are more desirable, as these temperatures are readily achieved in open outdoor cultivation systems without additional heating (Moheimani and Borowitzka, 2007).

Cyanobacterial tolerance to exogenous FFAs was previously investigated (Ruffing and Trahan, submitted). When a cytotoxic unsaturated FFA, α-linolenic acid, was exogenously added to cultures of *S. elongatus* PCC 7942 and *Synechococcus* sp. PCC 7002, *S. elongatus* PCC 7942 demonstrated a higher tolerance. At 5 μM of α-linolenic acid, *S. elongatus* PCC 7942 had minimal growth inhibition while *Synechococcus* sp. PCC 7002 growth was reduced by nearly 95%. Unexpectedly, cyanobacterial tolerance to intracellular FFA production led to inverse results, with *Synechococcus* sp. PCC 7002 having improved tolerance over *S. elongatus* PCC 7942. The improved FFA production tolerance of *Synechococcus* sp. PCC 7002 may be related to differences in membrane composition between these two strains. *Synechococcus* sp. PCC 7002 has an elevated amount of polyunsaturated membrane FAs as compared to *S. elongatus* PCC 7942 (unpublished data). The enhanced membrane viscosity associated with a lower degree of membrane saturation may allow for increased FFA diffusion across the cell membrane and therefore higher extracellular FFA concentrations. This proposed mechanism of FFA excretion also supports the aforementioned hypothesis for improved FFA tolerance at lower temperatures. The change in photosynthetic pigments in response to high FFA production also differed for the *Synechococcus* sp. PCC 7002 strains in comparison with the *S. elongatus* PCC 7942 strains. FFA-producing strains of *S. elongatus* PCC 7942 showed selective degradation of chlorophyll-*a* pigment in response to FFA production (Ruffing and Jones, 2012), while *Synechococcus* sp. PCC 7002 strains had reduced levels of both chlorophyll-*a* and the light-harvesting phycobiliproteins, phycocyanin, and allophycocyanin, during FFA production at 38°C. The degradation of phycobiliproteins is a common stress response among cyanobacteria (Collier and Grossman, 1994); however, the selective degradation of chlorophyll-*a* in FFA-producing strains of *S. elongatus* PCC 7942 appears to be an unconserved response.

In addition to differences in FFA stress response, *Synechococcus* sp. PCC 7002 also displayed different responses to the genetic manipulations of *aas/fadD* knockout, thioesterase expression, and RuBisCO expression. While Aas from *S. elongatus* PCC 7942 and FadD from *Synechococcus* sp. PCC 7002 are homologs (58.4% amino acid identity), *aas* knockout in SE01 led to the accumulation of 43 mg/L of excreted FFAs, yet *fadD* knockout in S01 yielded only 5 mg/L of excreted FFAs. These results may indicate that *S. elongatus* PCC 7942 has a naturally higher rate of membrane degradation and FFA recycling compared to *Synechococcus* sp. PCC 7002. In the *S. elongatus* PCC 7942 strains SE02 and SE03, both ‘TesA’ and ‘Fat1’ thioesterases had similar activities, generating nearly equivalent levels of excreted FFA (Ruffing, 2013a). In the analogous *Synechococcus* sp. PCC 7002 strains S02 and S03, however, only ‘TesA’ showed significant activity. Some improvement in Fat1 activity was achieved by using a truncated form, tFat1, with deletion of the predicted chloroplast-targeting signal. The overall low activity of ‘Fat1’ and ‘tFat1’ in S03 and S05 may be caused by reduced expression levels or possibly unidentified regulatory mechanisms. Lastly, no improvement in FFA production was achieved with RuBisCO expression in the FFA-producing strains of *S. elongatus* PCC 7942, while *Synechococcus* sp. PCC 7002 strains showed more than a threefold improvement in FFA production with heterologous *rbcLS* expression from the *psbA1* promoter of *S. elongatus* PCC 7942. This difference may indicate that carbon fixation is rate-limiting for *Synechococcus* sp. PCC 7002 but not for *S. elongatus* PCC 7942. Another possible explanation is that amino acid differences in RbcLS from *S. elongatus* PCC 7942 compared to that of *Synechococcus* sp. PCC 7002 led to improved activity in *Synechococcus* sp. PCC 7002. RbcL and RbcS from these two cyanobacteria show 86.2 and 68.81% identity in their amino acid sequences, indicating that these are highly similar proteins but not identical. Lastly, native *rbcLS* activity is post-transcriptionally regulated; thus, overexpression of native *rbcLS* in *S. elongatus* PCC 7942 did not improve carbon fixation, but the activity of *rbcLS* from *S. elongatus* PCC 7942 in the *Synechococcus* sp. PCC 7002 host was not regulated and therefore able to improve carbon flux and FFA biosynthesis. Heterologous RuBisCO expression was also shown to improve biofuel production by twofold in an isobutyraldehyde-producing strain of *S. elongatus* PCC 7942 (Atsumi et al., 2009). Codon usage in *S. elongatus* PCC 7942 and *Synechococcus* sp. PCC 7002 is very similar, with an average difference of 0.418 in percent usage for each codon sequence; this difference is slightly higher for the codons used in *rbcLS* (a difference of 13.3), suggesting that further improvement may be achieved with codon optimization. Regardless of the underlying mechanisms for improved FFA tolerance and production, *Synechococcus* sp. PCC 7002 was shown to be a superior host for FFA biosynthesis compared to *S. elongatus* PCC 7942. This work highlights the importance of host selection in metabolic engineering efforts, and since host responses are difficult to predict *a priori*, screening and testing of potential hosts remains a critical step in cyanobacterial strain development.

To date, three model cyanobacterial strains have been genetically engineered for FFA production: *Synechococcus* sp. PCC 7002 (this study), *S. elongatus* PCC 7942 (Ruffing and Jones, 2012; Ruffing, 2013a), and *Synechocystis* sp. PCC 6803 (Liu et al., 2011). These three strains are model hosts for biofuel production, as they have available genome sequences and established genetic tools and protocols for genetic manipulation. A comparison of these three strains with regard to their FFA tolerance, FFA production, and other desirable strain traits is presented in Table 2. While engineered *Synechocystis* sp. PCC 6803 strains yielded the highest FFA productivity and final FFA concentration, the genetic modifications in these strains (i.e., deletion of surface layer proteins in the cell wall) may weaken the integrity of the host strain and its ability to survive under adverse conditions, which may be experienced in large-scale production systems. Moreover, as the genetic manipulations differ between the engineered strains of *Synechocystis* sp. PCC 6803 and those of *Synechococcus* sp. PCC 7002 and *S. elongatus* PCC 7942, this direct comparison must be viewed with caution. *Synechocystis* sp. PCC 6803 also has the highest tolerance of exogenous FFA, yet the results of this study clearly demonstrate that exogenous FFA tolerance does not directly correlate with tolerance to intracellular FFA biosynthesis. When comparing inherent strain properties of doubling time, high light tolerance, high salt tolerance, and temperature tolerance, *Synechococcus* sp. PCC 7002 is superior to the other two cyanobacteria. These basic strain traits, which can be difficult to engineer into a host, make *Synechococcus* sp. PCC 7002 a beneficial host for cyanobacterial biofuel production, and this study provides direct evidence of the advantages afforded by this host, encouraging further investigation and development of *Synechococcus* sp. PCC 7002 for biofuel production.

**Table 2:** **Comparison of FFA production and host strain traits of three model cyanobacterial strains**.

Property	*Synechococcus* sp. PCC 7002	*S. elongatus* PCC 7942	*Synechocystis* sp. PCC 6803
FFA productivity (mg/L/h)^a^	0.273 (This study)	0.103 (Ruffing and Jones, 2012)	0.438 (Liu et al., 2011)
Final FFA concentration (mg/L)	131 (This study)	49.3 (Ruffing and Jones, 2012)	197 (Liu et al., 2011)
Exogenous FFA tolerance	Saturated FFA: no growth inhibition, polyunsaturated FFA: tolerant to <1 μM before growth is inhibited (Ruffing and Trahan, submitted)	Saturated FFA: no growth inhibition, polyunsaturated FFA: tolerant to 5 μM before growth is inhibited (Ruffing and Trahan, submitted)	Saturated FFA: no growth inhibition, polyunsaturated FFA: tolerant to 25 μM before growth is inhibited (Ruffing and Trahan, submitted)
Doubling time (h)	2.6–4 (Sakamoto and Bryant, 1997; Ludwig and Bryant, 2012)	5–6 (Kondo et al., 1997)	6 (Tu et al., 2004)
Light tolerance	Can grow under 4,500 μmol photons m^−2^ s^−1^ (Nomura et al., 2006)	Significant photodamage occurs with light intensities of 500–1000 μmol photons m^−2^ s^−1^ (Clarke et al., 1995; Kulkarni and Golden, 1995)	At 900 μmol photons m^−2^ s^−1^, rate of photodamage = rate of repair (Allakhverdiev and Murata, 2004)
Salt tolerance	1.7 M NaCl (Batterton and Baalen, 1971)	0.5 M NaCl (Fulda et al., 1999)	1.2 M NaCl (Fulda et al., 1999)
Temperature tolerance	Optimum = 34–38°C [this study (Ludwig and Bryant, 2012)], maximum growth temperature not determined	Optimum = 30–35°C (Mori et al., 2002), maximum growth temperature not determined	Optimum = 30°C, cannot grow above 43°C (Inoue et al., 2001)

*^a^FFA productivity calculated by taking the final concentration of excreted FFA concentration and dividing by the total time of cultivation*.

## Author Contributions

Anne M. Ruffing conceived the study, conducted all experimental work and data analysis, and drafted the manuscript.

## Conflict of Interest Statement

The author declares that the research was conducted in the absence of any commercial or financial relationships that could be construed as a potential conflict of interest.

## Supplementary Material

The Supplementary Material for this article can be found online at http://www.frontiersin.org/Journal/10.3389/fbioe.2014.00017/abstract
