# Supplementary material for: Improved Free Fatty Acid Production in Cyanobacteria with Synechococcus sp. PCC 7002 as Host
Source: Front Bioeng Biotechnol. 2014 May 26;2:17. doi: 10.3389/fbioe.2014.00017 (PMC4126656; doi:10.3389/fbioe.2014.00017)
Supplement: Supplementary file 1 [file Data_Sheet1.DOCX]

**Supplemental Information**

This document provides supplemental information for the article *Improved Free Fatty Acid Production in Cyanobacteria with Synechococcus sp. PCC 7002 as Host*. Supplemental information includes a schematic of the metabolic pathways engineered in this work (Figure S1), a comparison of codon usage in the *rbcLS* operon (Table S1), plasmids and primers used in strain construction (Tables S2 and S3) and select plasmid maps (Figure S2).


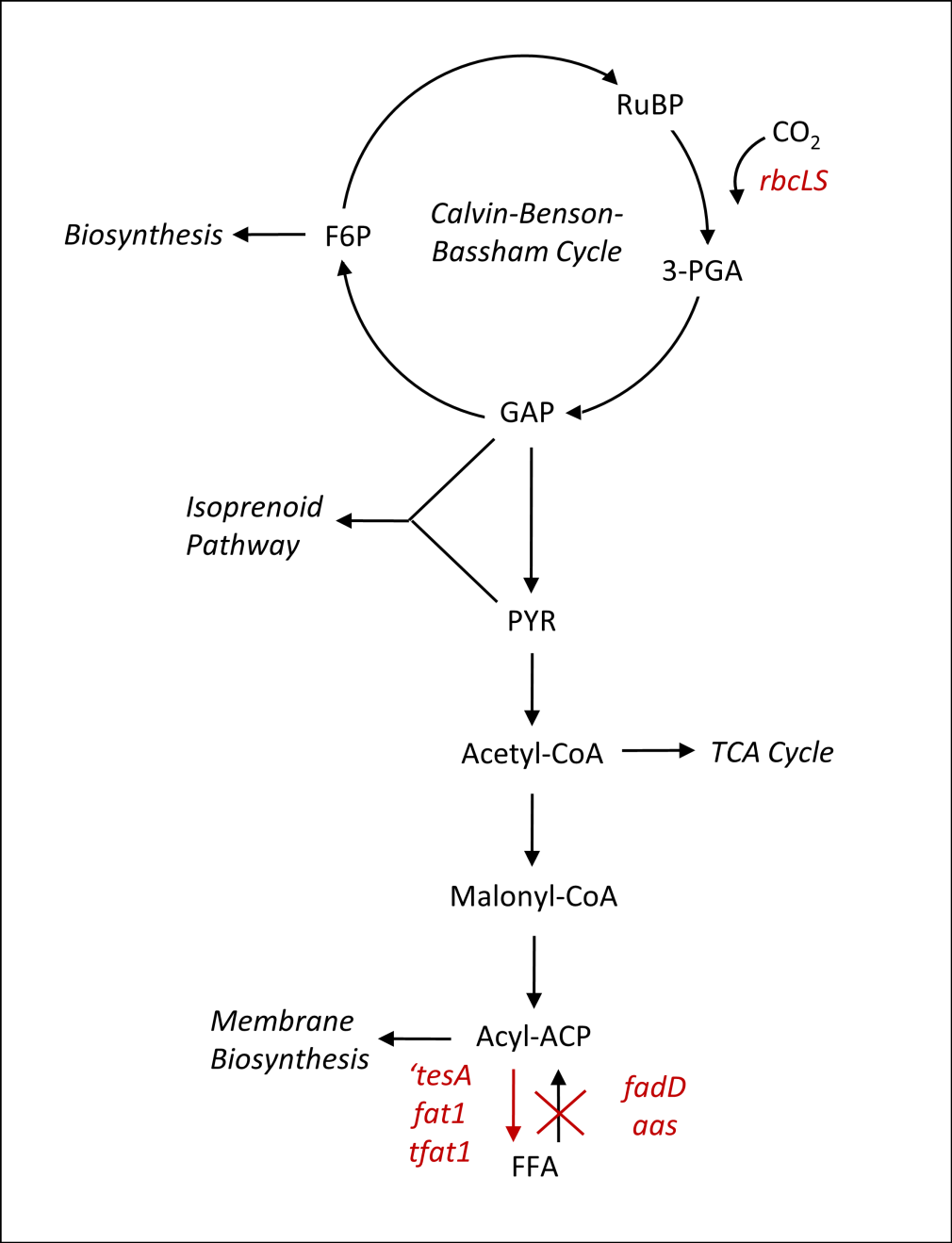


**Figure S1.** Schematic of the metabolic pathways involved in free fatty acid production in cyanobacteria. Metabolic engineering targets are highlighted in red and include gene knockout of the acyl-ACP synthase/long-chain-fatty-acid CoA ligase (*fadD/aas*), expression of a thioesterase (*‘tesA, fat1, tfat1*), and overexpression of RuBisCO (*rbcLS*). This figure is adapted from ([Ruffing, 2013](#_ENREF_1)).

**Table S1.** Comparison of codon usage in the *rbcLS* operon in *Synechococcus* sp. PCC 7002 and *Synechococcus elongatus* PCC 7942.

| **Amino Acid** | **Codon** | **7002** | | **7942** | | **%usage difference** |
| --- | --- | --- | --- | --- | --- | --- |
|  |  | **Number** | **% usage** | **Number** | **% usage** |  |
| Ala | GCG | 9 | 20.9 | 10 | 21.3 | 0.3 |
|  | GCA | 7 | 16.3 | 8 | 17.0 | 0.7 |
|  | GCT | 19 | 44.2 | 21 | 44.7 | 0.5 |
|  | GCC | 8 | 18.6 | 8 | 17.0 | 1.6 |
| Cys | TGT | 8 | 57.1 | 2 | 18.2 | 39.0 |
|  | TGC | 6 | 42.9 | 9 | 81.8 | 39.0 |
| Asp | GAT | 15 | 42.9 | 9 | 25.0 | 17.9 |
|  | GAC | 20 | 57.1 | 27 | 75.0 | 17.9 |
| Glu | GAG | 3 | 7.5 | 13 | 32.5 | 25.0 |
|  | GAA | 37 | 92.5 | 27 | 67.5 | 25.0 |
| Phe | TTT | 4 | 14.8 | 6 | 18.8 | 3.9 |
|  | TTC | 23 | 85.2 | 26 | 81.3 | 3.9 |
| Gly | GGG | 3 | 6.3 | 4 | 8.2 | 1.9 |
|  | GGA | 1 | 2.1 | 0 | 0.0 | 2.1 |
|  | GGT | 37 | 77.1 | 24 | 49.0 | 28.1 |
|  | GGC | 7 | 14.6 | 21 | 42.9 | 28.3 |
| His | CAT | 0 | 0.0 | 2 | 10.5 | 10.5 |
|  | CAC | 15 | 100.0 | 17 | 89.5 | 10.5 |
| Ile | ATA | 0 | 0.0 | 0 | 0.0 | 0.0 |
|  | ATT | 3 | 14.3 | 1 | 3.4 | 10.8 |
|  | ATC | 18 | 85.7 | 28 | 96.6 | 10.8 |
| Lys | AAG | 19 | 67.9 | 12 | 38.7 | 29.1 |
|  | AAA | 9 | 32.1 | 19 | 61.3 | 29.1 |
| Leu | TTG | 4 | 7.3 | 11 | 22.0 | 14.7 |
|  | TTA | 5 | 9.1 | 0 | 0.0 | 9.1 |
|  | CTG | 13 | 23.6 | 25 | 50.0 | 26.4 |
|  | CTA | 0 | 0.0 | 0 | 0.0 | 0.0 |
|  | CTT | 5 | 9.1 | 1 | 2.0 | 7.1 |
|  | CTC | 28 | 50.9 | 13 | 26.0 | 24.9 |
| Met | ATG | 12 | 100.0 | 16 | 100.0 | 0.0 |
| Asn | AAT | 4 | 18.2 | 2 | 11.8 | 6.4 |
|  | AAC | 18 | 81.8 | 15 | 88.2 | 6.4 |
| Pro | CCG | 3 | 10.0 | 10 | 33.3 | 23.3 |
|  | CCA | 0 | 0.0 | 1 | 3.3 | 3.3 |
|  | CCT | 11 | 36.7 | 7 | 23.3 | 13.3 |
|  | CCC | 16 | 53.3 | 12 | 40.0 | 13.3 |
| Gln | CAG | 8 | 38.1 | 6 | 31.6 | 6.5 |
|  | CAA | 13 | 61.9 | 13 | 68.4 | 6.5 |
| Arg | AGG | 0 | 0.0 | 0 | 0.0 | 0.0 |
|  | AGA | 0 | 0.0 | 0 | 0.0 | 0.0 |
|  | CGG | 3 | 8.6 | 3 | 8.6 | 0.0 |
|  | CGA | 0 | 0.0 | 0 | 0.0 | 0.0 |
|  | CGT | 20 | 57.1 | 18 | 51.4 | 5.7 |
|  | CGC | 12 | 34.3 | 14 | 40.0 | 5.7 |
| Ser | AGT | 1 | 4.3 | 0 | 0.0 | 4.3 |
|  | AGC | 4 | 17.4 | 7 | 26.9 | 9.5 |
|  | TCG | 1 | 4.3 | 12 | 46.2 | 41.8 |
|  | TCA | 0 | 0.0 | 0 | 0.0 | 0.0 |
|  | TCT | 12 | 52.2 | 2 | 7.7 | 44.5 |
|  | TCC | 5 | 21.7 | 5 | 19.2 | 2.5 |
| Thr | ACG | 2 | 5.0 | 5 | 15.2 | 10.2 |
|  | ACA | 1 | 2.5 | 0 | 0.0 | 2.5 |
|  | ACT | 18 | 45.0 | 3 | 9.1 | 35.9 |
|  | ACC | 19 | 47.5 | 25 | 75.8 | 28.3 |
| Val | GTG | 2 | 5.0 | 12 | 37.5 | 32.5 |
|  | GTA | 10 | 25.0 | 0 | 0.0 | 25.0 |
|  | GTT | 24 | 60.0 | 5 | 15.6 | 44.4 |
|  | GTC | 4 | 10.0 | 15 | 46.9 | 36.9 |
| Trp | TGG | 10 | 100.0 | 11 | 100.0 | 0.0 |
| Tyr | TAT | 5 | 21.7 | 2 | 10.0 | 11.7 |
|  | TAC | 18 | 78.3 | 18 | 90.0 | 11.7 |
| Stop | TGA | 0 | 0.0 | 0 | 0.0 | 0.0 |
|  | TAG | 0 | 0.0 | 0 | 0.0 | 0.0 |
|  | TAA | 2 | 100.0 | 2 | 100.0 | 0.0 |

**Table S2.** Plasmids used and constructed in this study.

| **Plasmid** | **Description** | **Reference** |
| --- | --- | --- |
| pSE15 | Modified pAM2991 with *S. elongatus* PCC 7942 neutral site I (NSI) homologous regions replaced with ~ 1 kb regions of homology to upstream and downstream regions of the acyl-ACP synthetase/long-chain-fatty-acid CoA ligase (*aas,* Synpcc7942_0918) | ([Ruffing and Jones, 2012](#_ENREF_2)) |
| pSE16 | Modified pSE15 with a truncated *E. coli* thioesterase (*‘tesA*) inserted downstream of P_trc_ | ([Ruffing and Jones, 2012](#_ENREF_2)) |
| pSE18 | Modified pSE15 with *C. reinhardtii* acyl-ACP thioesterase (*fat1*) and the large and small subunits of RuBisCO (*rbcLS*) from *S. elongatus* PCC 7942 inserted after P_trc_ to form the synthetic operon P_trc_-*fat1*-*rbcLS* | ([Ruffing, 2013](#_ENREF_1)) |
| pSE20 | Modified pSE15 with *C. reinhardtii* acyl-ACP thioesterase (*fat1*) inserted after P_trc_ and the large and small subunits of RuBisCO (*rbcLS*) from *S. elongatus* PCC 7942 inserted after the *psbAI* promoter to form the synthetic operon P_trc_-*fat1*-P*_psbAI_*-*rbcLS* | ([Ruffing, 2013](#_ENREF_1)) |
| pS12 | Modified pSE15 with *S. elongatus* PCC 7942 NSI homologous regions replaced with ~ 500 bp regions of homology to upstream and downstream regions of the acyl-ACP synthetase/long-chain-fatty-acid CoA ligase (*fadD,* SYNPCC7002_A0675) | This study |
| pS13 | Modified pS12 with a truncated *E. coli* thioesterase (*‘tesA*) inserted downstream of P_trc_ | This study |
| pS14 | Modified pS12 with *C. reinhardtii* acyl-ACP thioesterase (*fat1*) inserted downstream of P_trc_ | This study |
| pS17 | Modified pS12 with truncated *C. reinhardtii* acyl-ACP thioesterase (*tfat1*) inserted downstream of P_trc_ | This study |
| pS18 | Modified pS12 with a truncated *E. coli* thioesterase (*‘tesA*) inserted downstream of P_trc_ with AatII restriction site inserted downstream of *‘tesA* | This study |
| pS19 | Modified pS18 with large and small subunits of RuBisCO (*rbcLS*) from *S. elongatus* PCC 7942 inserted downstream of *‘tesA* | This study |
| pS20 | Modified pS18 with the *psbAI* promoter from *S. elongatus* PCC 7942 inserted after the truncated thioesterase (*‘tesA*) and introduction of BsrGI restriction site added on the reverse primer | This study |
| pS21 | Modified pS20 with the large and small subunits of RuBisCO (*rbcLS*) from *S. elongatus* PCC 7942 inserted after the *psbAI* promoter | This study |
| pSB | Modifed pSA ([Ruffing, 2013](#_ENREF_1)) with neutral site II (NSII) homologous regions replaced by desaturase B (*desB*) homologous regions | This study |

**Table S3.** Primers used in this study. Restriction sites are in italics.

| **Name** | **Sequence** | **Description** |
| --- | --- | --- |
| fadD3F | GATCTA*AGATCT*GCGATCCGAATGGCGGAATCTTCG | Used to clone the 3’ homologous region for *fadD* knockout in pS12 |
| fadD3R | CTACTT*CTCGAG*CGCACCAGATTATCGCCCACTTTCA |  |
| fadD5F | GCACTG*CTTAAG*AAAATATCGAGGTAGCGGTCTAAAACC | Used to clone the 5’ homologous region for *fadD* knockout in pS12 |
| fadD5R | CTTGAG*ACTAGT*GCCGAAATCATGGCTACAATCCTAC |  |
| tesAF | GTGATG*GAATTC*GCAGCGGACACGTTATTGATTCTGG | Used to clone *‘tesA* from pSE16 |
| tesAR | CGAGTC*GGATCC*TTATGAGTCATGATTTACTAAAGGCTGC |  |
| tesAR2 | CGATTG*GGATCCGACGTC*TTATGAGTCATGATTTACTAAAGGCTGC | Used to insert AatII site on the 3’ end of *‘tesA* |
| desB5F | CTAGCT*GAGCTC*CTGATGAAAGACCGTCCACA | Used to clone the 5’ homologous region for *desB* knockout in pSB |
| desB5R | TGTGAC*GAGCTC*TCAGTTTCAAAAAGAGATTAACA |  |
| desB3F | TCAGTA*CCTAGG*GCTCCAAAAGCGTGACTAGAT | Used to clone the 3’ homologous region for *desB* knockout in pSB |
| desB3R | GTACAA*CCTAGG*ACGGGCCAGGCGATAGAGA |  |
| fat1F | GTACCA*GAATTC*ATGCGTCGGTTTGCCACGTTGAACGAGCA | Used to clone *fat1* from pSE20 |
| fat1R | TCTAAA*GAATTCGACGTC*TCACTGGGCCGCAGACAGCTCCGACAG |  |
| tfat1F | GCACCA*GAATTC*ATGGCAGCGGCGGTGGTTCAGGA | Used to remove the chloroplast targeting signal from *fat1* |
| rbcLSF1 | GTCATT*GACGTC*CTAGGGAGAGACGACATGCCCAAGA | Used to clone *rbcLS* from pSE18 |
| rbcLSR1 | GTCACT*GACGTC*TTAGTAGCGGCCAGGACGATGAACG |  |
| PpsbF | GCACTC*GACGTC*CTGGATTTAGCGTCTTCTAATC | Used to clone P*_psbAI_* from pSE20 |
| PpsbR | CATTGT*GACGTCTGTACA*CTTGAGGTTGTAAAGGGCAAG |  |
| rbcLSF2 | GTCATC*TGTACA*CT*AGGGAGAGA*CGACATGCCCAAGA | Used to clone *rbcLS* from pSE20 |
| rbcLSR2 | GTCACT*TGTACA*TTAGTAGCGGCCAGGACGATGAACG |  |


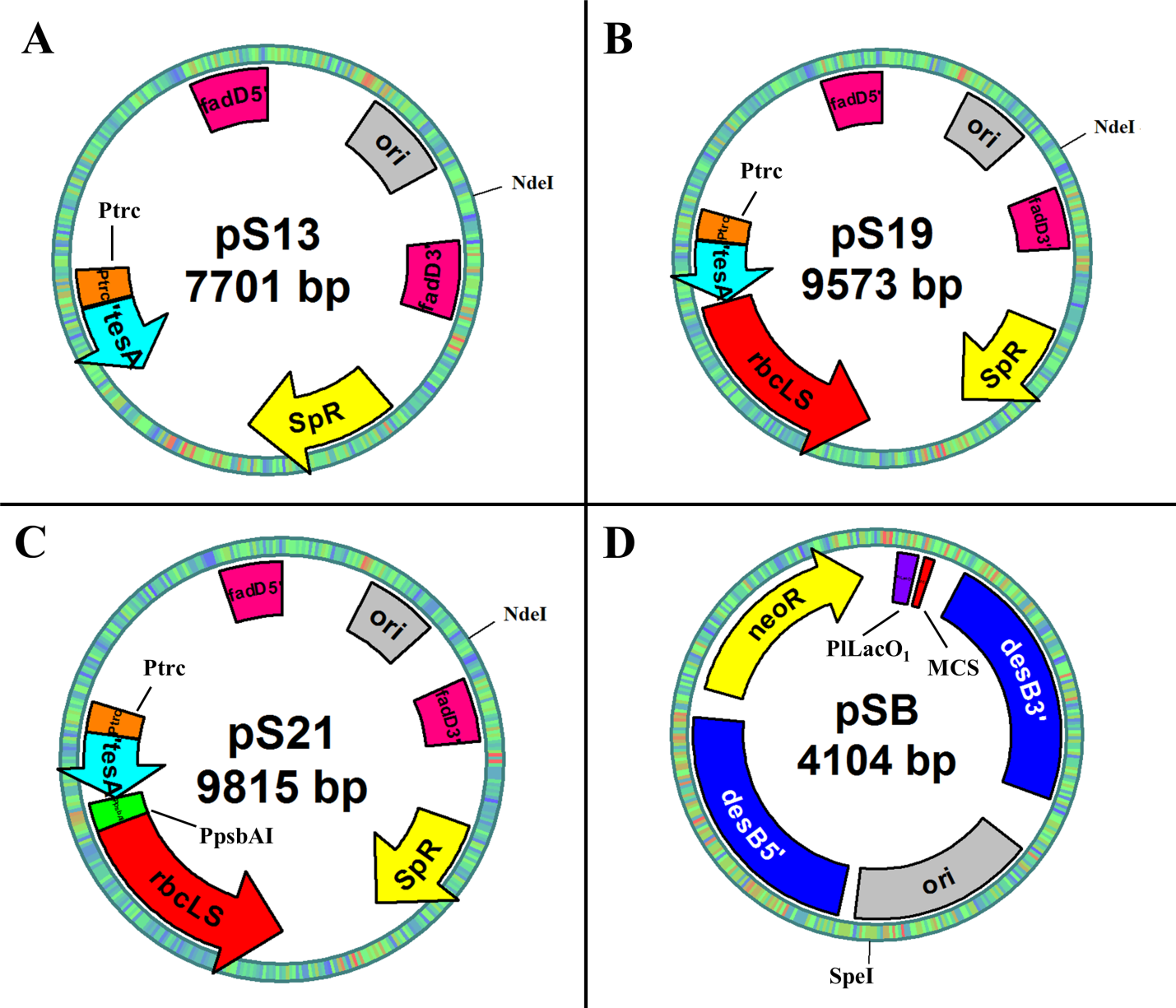


**Figure S2.** Plasmid maps for select plasmids constructed in this study. pS13 for construction of strain S02 (A). pS19 for construction of strain S06 (B). pS21 for construction of strain S07 (C). pSB for construction of 7002ΔdesB (D). The restriction site for plasmid linearization is shown in each map (*Nde*I or *Spe*I). Abbreviations: MCS – multicloning site, neoR – neomycin/kanamycin resistance, ori – origin of replication, SpR – spectinomycin resistance.

**References**

Ruffing, A.M. (2013). Borrowing genes from *Chlamydomonas reinhardtii* for free fatty acid production in engineered cyanobacteria. *Journal of Applied Phycology* 25**,** 1495-1507.

Ruffing, A.M., and Jones, H.D.T. (2012). Physiological effects of free fatty acid production in genetically engineered *Synechococcus elongatus* PCC 7942. *Biotechnology and Bioengineering* 109**,** 2190-2199. doi: 10.1002/bit.24509.
